# Diagnostic value of inflammatory indexes combined with myocardial enzymes for aneurysmal subarachnoid hemorrhage in patients with intracranial aneurysms

**DOI:** 10.1016/j.ibneur.2025.08.023

**Published:** 2025-08-30

**Authors:** Haihong Zhang, Siyuan Dong, Qian Liu, Hongguang Wang

**Affiliations:** a Clinical College of Neurology, Neurosurgery and Neurorehabilitation, Tianjin Medical University, Tianjin, China; b Department of Neurosurgery, Tianjin Huanhu Hospital, Tianjin, China; c Xinjiang Medical University Fifth Affiliated Hospital, Xinjiang, China

**Keywords:** Inflammatory indexes, Myocardial enzymes, Intracranial aneurysm, Aneurysmal subarachnoid hemorrhage, Diagnostic value

## Abstract

**Objective:**

The purpose of the study is to evaluate the diagnostic ability of inflammatory indexes alone and in combination with myocardial enzymes in the prediction of aneurysmal subarachnoid hemorrhage (aSAH).

**Materials and methods:**

This is a 1:1 age- and sex-matched case-control study in Tianjin, China. 226 patients aged > 18 years divided into aSAH group and unruptured intracranial aneurysm (UIA) group. Logistic regression models assessed associations between inflammatory indexes/myocardial enzymes and aSAH. Receiver operating characteristic (ROC) curves evaluated their sensitivity/specificity for aSAH.

**Results:**

Higher neutrophil-to-lymphocyte ratio (NLR) (OR: 1.130, 95 % CI: 1.074, 1.188), monocyte-to-lymphocyte ratio (MLR) (OR: 11.144, 95 % CI: 3.071, 40.437), platelet-to-lymphocyte ratio (PLR) (OR: 1.006, 95 % CI: 1.003, 1.008), systemic immune-inflammatory index (SII) (OR: 1.000, 95 % CI: 1.000, 1.001) and systemic inflammation response index (SIRI) (OR: 1.228, 95 % CI: 1.112, 1.357), myocardial enzymes lactate dehydrogenase (LDH) (OR: 1.008, 95 % CI: 1.003, 1.014), creatine kinase (CK) (OR: 1.002, 95 % CI: 1.000, 1.004), and α-hydroxybutyrate dehydrogenase (HBDH) (OR: 1.010, 95 % CI: 1.002, 1.018) were all significantly associated with the presence of aSAH. The diagnostic value of inflammatory indexes (NLR-PLR-MLR-SII-SIRI) and myocardial enzymes (LDH-HBDH-CK-CKMB) gave an AUC of 0.771 and 0.662 (all *P*-value < 0.001), respectively. Combined use of these indexes achieved an AUC of 0.755 (*P*-value< 0.001), suggesting potential value as a diagnostic adjunct for aSAH.

**Conclusions:**

The results suggest that higher inflammatory indexes and myocardial enzymes were associated with the presence of aSAH. The combination of inflammatory indexes and myocardial enzymes may serve as a diagnostic complement for identifying aSAH in patients with IA.

## Introduction

Intracranial aneurysm (IAs) is defined as an abnormal expansion in the intracranial arteries, which may lead to aneurysm rupture, affecting approximately 3–5 % of the general population (Ding et al., 2021). However, only < 2 % of cases progress to aneurysmal subarachnoid hemorrhage (aSAH), indicating that rupture occurs in a small subset of unruptured intracranial aneurysm (UIA) patients. Despite this, aSAH remains a life-threatening condition, with mortality rates as high as 57 % within six months of rupture (Kim et al., 2019). Given the severe consequences of aneurysm rupture, investigating the mechanisms underlying aneurysm formation, growth, and rupture is crucial for improving clinical outcomes.

Magnetic resonance imaging (MRI)-derived circumferential aneurysm wall enhancement has been linked to active vascular inflammation and macrophage accumulation, suggesting its utility in assessing rupture risk. Nevertheless, the cell-specific pathological changes in human IA tissues and the dysregulated inflammatory pathways driving rupture remain insufficiently understood (Etminan and Rinkel, 2016).

Previous studies have established a significant association between IA rupture and inflammatory/immune mechanisms. For instance, bioinformatics analyses have demonstrated a strong correlation between dysregulated inflammatory pathways, immune system activation, and aneurysm rupture (Tian et al., 2024).

Targeting inflammatory pathways may represent a promising therapeutic strategy to mitigate rupture risk and reduce the incidence of aSAH (Mirbagheri et al., 2024). Peripheral blood biomarkers offer a clinically accessible and economically viable approach for risk stratification, as elevated inflammatory markers consistently correlate with disease progression across multiple pathological conditions. Established hematological indices, including the neutrophil-to-lymphocyte ratio (NLR), monocyte-to-lymphocyte ratio (MLR), and platelet-to-lymphocyte ratio (PLR), have emerged as reliable proxies for systemic inflammatory status (Zhu et al., 2023). More recently, novel composite indices—the systemic immune-inflammatory index (SII) and systemic inflammation response index (SIRI)—have gained recognition as robust, cost-effective inflammatory biomarkers. A large-scale prospective cohort study demonstrated SIRI’s significant association with poor long-term functional outcomes in aSAH patients (Li et al., 2024). Furthermore, postoperative day 3 measurements of SII and SIRI were identified as independent predictors of unfavorable prognosis in aSAH cases (Wang et al., 2024). Although these inflammatory indices share common components (e.g., neutrophils, lymphocytes), each captures distinct aspects of immune response: NLR primarily reflects the balance between innate and adaptive immunity, which is particularly sensitive for assessing acute inflammatory responses and stress levels. MLR focuses on chronic inflammation and immune regulation, with special relevance to monocyte-mediated endothelial dysfunction in vascular diseases. PLR uniquely integrates information about both coagulation and immune systems, making it valuable for evaluating thromboinflammatory conditions. SII provides a more comprehensive assessment by combining thrombotic and inflammatory pathways, particularly useful in oncology and cardiovascular research. SIRI emphasizes overall myeloid cell activation status, offering a broader perspective on systemic inflammation (Bailey-Whyte et al., 2023). Therefore, these inflammatory indices are routinely incorporated as a comprehensive panel in research analyses.

Myocardial enzymes, including creatine kinase (CK), its myocardial-specific isoenzyme CK-MB, and lactate dehydrogenase (LDH), represent established biochemical markers of myocardial injury. The clinical value of the LDH isoform α-hydroxybutyrate dehydrogenase (HBDH) has also come into focus (Naidech et al., 2005). Persistent observations of myocardial biomarker elevation in aSAH have been documented, yet the underlying pathophysiological mechanisms remain incompletely elucidated. Notably, post-procedural elevation of cardiac troponin I (TnI) following ruptured IA occlusion has been shown to predict major adverse cardiac events within one year, suggesting potential involvement of neurocardiac injury - a phenomenon of brain-mediated myocardial dysfunction (Lee et al., 2022). Interestingly, emerging evidence indicates that ruptured aneurysm size independently predicts the degree of myocardial biomarker elevation, with CK-MB demonstrating particular promise as a complementary diagnostic marker for improving aSAH risk stratification (Chen et al., 2022). These seemingly paradoxical findings highlight the need for mechanistic studies to clarify potential causal relationships.

Of particular significance is myocarditis, an inflammatory cardiomyopathy characterized by myocardial infiltration and edema. The NF-κB signaling pathway appears to play a central role in mediating this inflammatory cascade, driving the production of pro-inflammatory cytokines and chemokines (Ullah et al., 2022). This inflammatory milieu is reflected in the strong correlation observed between elevated cardiac enzymes and systemic inflammatory markers, including high-sensitivity C-reactive protein (hs-CRP) and serum amyloid A (SAA), suggesting a potential mechanistic link between myocardial injury and systemic inflammation (Ullah et al., 2022). Furthermore, while elevated SIRI and SII showed significant associations with increased mortality in acute myocardial infarction, these relationships were attenuated following multivariate adjustment for confounding factors (Marchi et al., 2024). This observation implies that while systemic inflammation may contribute to adverse cardiac outcomes, its predictive value may be influenced by other clinical variables (Marchi et al., 2024).

In this 1:1 matched case-control study, we aimed to investigate the association between peripheral blood immune cell-derived inflammatory indices (NLR, MLR, PLR, SII, and SIRI) and myocardial injury biomarkers (LDH, HBDH, CK, and CK-MB) with the presence of aSAH. Furthermore, we sought to evaluate the diagnostic performance of these systemic inflammatory markers, both individually and in combination with myocardial enzymes, through receiver operating characteristic (ROC) curve analysis to aid in identifying aSAH.

## Materials and methods

### Study population

**Fig. 1:**
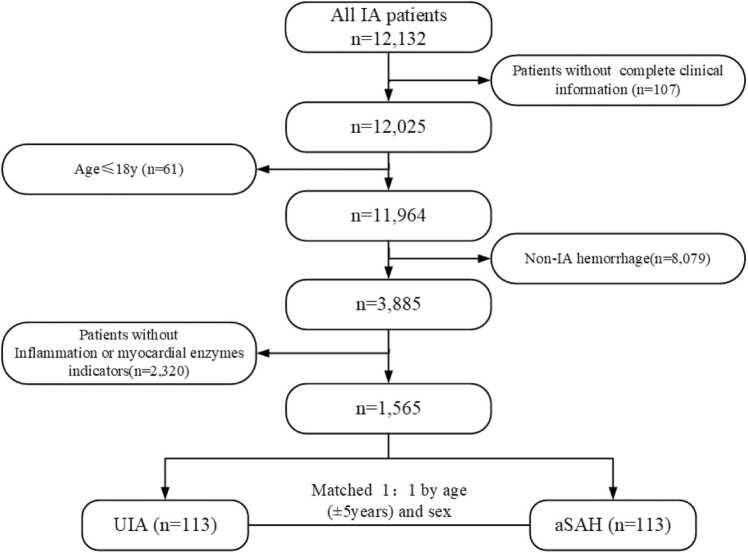
Technology roadmap of the study.

This study was a 1: 1 age- (±5 years) and gender-matched case-control study. The participants were recruited from the Department of Neurosurgery, Tianjin Huanhu Hospital for this study. The case-control study was conducted in the subjects aged 18 years or older and divided into two different groups: aSAH and UIA. In total, 12,132 patients were identified from January 2016 to January 2024, and the data were accessed for research purposes in 01/09/2024. A total of 10,567 patients were excluded from this analysis based on the following criteria: patients without complete clinical information (*n* = 107); patients aged 18 years or younger (*n* = 61); patients diagnosed with non-IA hemorrhage (*n* = 8079); and patients without inflammation and myocardial enzymes indicators (*n* = 2320). Finally, a total of 1565 individuals were included in this study. There was a total of 113 patients with UIA in this population, and aSAH were age- and sex-matched (Fig. 1). Information that could identify individual participant was fully anonymized during or after data collection. The study protocol was approved by the Ethics Committee of Tianjin Huanhu Hospital (*No*. 2024–190), and all participants provided handwritten informed consent. All procedures in this study were conducted according to the Declaration of Helsinki.

### The clinical diagnosis of IA

All patients diagnosed with aSAH and UIA undergo computed tomography angiography (CTA), Magnetic Resonance Angiography (MRA), and Digital Subtraction Angiography (DSA).

### Laboratory index detection

All blood samples were obtained within 24 h post-admission and before any treatment initiation, and the blood samples were used for immediate testing of the complete blood count. The measurements of immunity markers, including counts of white blood cells (WBC), neutrophils (NEU), platelets (PLT), lymphocytes (LYM), monocytes (MON), and myocardial enzyme spectrum, including LDH, HBDH, CK, and CK-MB were performed using a Siemens ADVIA 2400 automatic biochemical analyzer (Berlin, Germany) with standard laboratory techniques.

NLR, MLR, PLR, PLT%, NEU%, LYM%, MON%, SII and SIRI were calculated as follows: NLR = neutrophil count/lymphocyte count, MLR = monocyte count/lymphocyte count, PLR = platelet count/lymphocyte count, PLT% = platelet count/WBC, NEU% = neutrophil count/WBC, LYM% = lymphocyte count/WBC, MON% = monocyte count/WBC, SII = platelet count*NLR, and SIRI = neutrophil count*MLR.

### Statistical analysis

Continuous variables were expressed as means ± SDs or medians (IQRs) depending on normality. Categorical variables were shown as frequencies (percentages). The normality of continuous variables was assessed using the Skewness-Kurtosis test. Non-normal data distributions were normalized with log-transformed. A paired *t*-test was conducted for continuous variables and the *chi*-square test was used for categorical variables to compare the differences between groups of aSAH and UIA. Collinearity diagnostics, including assessments of variance inflation factor (VIF) and tolerance, were performed to rule out potential collinearity. A VIF value below 10 or a tolerance (the inverse of VIF) above 0.1 typically indicates the absence of problematic multicollinearity (Estraneo et al., 2020). Logistic regression analysis was performed to test the associations of immunologic parameters and myocardial enzyme spectrum with aSAH. Results were presented as odds ratios (ORs) and 95 % confidence intervals (CIs). Pearson’s correlation analysis and linear regression analysis were performed to explore the relationships between immunologic parameters and the myocardial enzyme spectrum. Results were presented as correlation coefficient and *β*-coefficients with *95 % CI*s, respectively. Model 1 was a crude model, Model 2 adjusted for age, sex, smoking, alcohol consumption, history of hypertension, diabetes, and heart disease. In order to avoid false positives, all reported *P*-values underwent false discovery rate (FDR) correction using the Benjamini-Hochberg procedure to account for multiple comparisons. Associations with Q-values < 0.05 were considered statistically significant after adjustment (Hayden et al., 2020). ROC curves were used to compare the diagnostic value of different variables, and the diagnostic ability of inflammatory indexes, myocardial enzymes and all these indexes were compared using the Delong’s test (Zhao et al., 2021). Outliers were identified as > 2 SD of the mean, and they were replaced by the respective mean values. Sensitivity analysis was conducted in the whole unmatched sample of 1565 participants to assess the robustness of the results. All analyses were performed using SPSS Version 25.0 (SPSS Inc., Chicago, IL, USA) and graphed using GraphPad Prism 9.0 and statistical software R (R version 3.4.3). A two-sided *P* value< 0.05 was considered to be statistically significant.

## Results

### Characteristics of the study population

As shown in Table 1, compared with the UIA group, aSAH patients had higher levels of WBC, neutrophil counts, monocyte counts, NEU%, NLR, MLR, PLR, SII, SIRI, LDH, HBDH, CK-MB, and CK (all *P*-values < 0.05). And aSAH patients had lower levels of lymphocyte counts, LYM% and MON% (all *P*-values < 0.05). There were no significant differences between the groups of aSAH and UIA with regard to age, sex, smoking status, alcohol drinking, hypertension, diabetes, heart disease, and platelet counts (all *P*-values > 0.05).

**Table 1:** Demographic and clinical characteristics of the participants (*n* = 226).

Characteristics	aSAH	UIA	*P-*value
*n* (%)	113 (50.0)	113 (50.0)	
Age, years	59.73 ± 11.68	59.41 ± 11.13	0.829
Female, *n* (%)	71 (62.80)	71 (62.80)	1.000
Smoking, *n* (%)	25 (22.10)	28 (24.80)	0.638
Drinking, *n* (%)	22 (19.50)	12 (10.60)	0.063
Hypertension, *n* (%)	75 (66.40)	77 (68.10)	0.777
Diabetes, *n* (%)	12 (10.60)	17 (15.00)	0.320
Heart disease, *n* (%)	11 (9.70)	17 (15.00)	0.226
WBC (×10^9^/L)	11.32 ± 4.34	8.12 ± 3.41	< 0.001
Platelet counts (×10^9^/L)	238.76 ± 73.03	227.66 ± 56.77	0.203
Neutrophil counts (×10^9^/L)	9.63 ± 4.38	5.98 ± 3.61	< 0.001
Lymphocyte counts (×10^9^/L)	1.18 ± 0.59	1.61 ± 0.75	< 0.001
Monocyte counts (×10^9^/L)^†^	0.47 ± 0.24	0.42 ± 0.16	< 0.001
PLT%	0.27 ± 0.07	0.26 ± 0.07	0.454
NEU%	83.20 ± 9.33	69.93 ± 14.21	< 0.001
LYM%	11.99 ± 7.53	22.94 ± 12.03	< 0.001
MON%	4.33 ± 2.08	5.54 ± 1.98	< 0.001
NLR	10.76 ± 8.12	5.50 ± 5.97	< 0.001
MLR	0.44 ± 0.24	0.32 ± 0.22	< 0.001
PLR	213.79 (154.67, 306.54)	142.70 (103.66, 225.51)	< 0.001
SII	2585.47 ± 2252.55	1259.52 ± 1490.68	< 0.001
SIRI	3.59 (1.80, 6.31)	1.15 (0.70, 2.49)	< 0.001
LDH (U/L)	224.12 ± 61.55	199.04 ± 51.80	0.001
HBDH (U/L)	139.82 ± 37.88	128.55 ± 31.84	0.016
CK-MB (U/L)	13.00 (10.00, 17.00)	12.00 (10.00, 15.00)	0.036
CK (U/L)	83.00 (50.00, 145.50)	62.00 (42.00, 102.50)	0.014

Continuous variables are presented as mean ± SD or median (25th, 75th percentiles) using the paired-*t* test; categorical variables are presented as *n* (%) using the *chi*-square test. CK, CK-MB, PLR, and SIRI were logarithmically transformed. aSAH, aneurysmal subarachnoid hemorrhage; UIA, unruptured intracranial aneurysm; WBC, White blood cell; PLT, Platelet counts; NEU, Neutrophil count; LYM, Lymphocyte counts; MON, Monocyte counts; NLR, Neutrophil to lymphocyte ratio; MLR, Monocyte to lymphocyte ratio; PLR, Platelet to lymphocyte ratio; SII, systematic inflammation index; SIRI, systematic inflammation response index; LDH, lactate dehydrogenase; HBDH, hydroxybutyrate dehydrogenase; CK-MB, creatine kinase-MB; CK, creatine kinase.

### Association between inflammatory indexes and myocardial enzymes with aSAH

Collinearity analyses were performed for intercorrelated inflammatory indexes (NLR, MLR, PLR, SII, SIRI). Results showed no collinearity among independent variables, with all variance VIFs < 10, as detailed in Supplementary Table 1. Table 2 shows the associations between inflammatory indexes and myocardial enzymes with aSAH. In the crude model, a higher level of WBC (*OR:* 1.281, *95 % CI*: 1.168, 1.405), a higher count of neutrophil (*OR:* 1.305, *95 % CI*: 1.190, 1.431), a lower count of lymphocyte (*OR:* 0.371, *95 % CI*: 0.236, 0.582), a higher NEU% (*OR:* 1.091, *95 % CI*: 1.063, 1.119), a lower LYM% (*OR:* 0.900, *95 % CI*: 0.872, 0.929), a lower MON% (*OR:* 0.745, *95 % CI*: 0.648, 0.856), a higher NLR (*OR:* 1.130, *95 % CI*: 1.074, 1.188), a higher MLR (*OR:* 11.144, *95 % CI*: 3.071, 40.437), a higher PLR (*OR:* 1.006, *95 % CI*: 1.003, 1.008), a higher SII (*OR:* 1.000, *95 % CI*: 1.000, 1.001), a higher SIRI (*OR:* 1.228, *95 % CI*: 1.112, 1.357), a higher LDH (*OR:* 1.008, *95 % CI*: 1.003, 1.014), a higher HBDH (*OR:* 1.010, *95 % CI*: 1.002, 1.018), and a higher CK (*OR:* 1.002, *95 % CI*: 1.000, 1.004) were all significantly associated with an increased risk of aSAH. After multivariate adjustment, similar results were observed except for the association of the count of monocytes (*OR:* 4.227, *95 % CI*: 1.022, 17.478), and level of CK (*OR:* 1.002, *95 % CI*: 1.000, 1.004) with aSAH. After Benjamini-Hochberg FDR adjustment, only monocyte counts failed to maintain statistical significance (*Q*-value =0.060 vs. threshold of 0.05), while all other inflammatory indices and myocardial enzymes demonstrated robust associations (all *Q*-value < 0.05).

**Table 2:** Associations between inflammatory indexes and myocardial enzymes with aSAH (*n=*226).

Parameters	Model 1			Model 2			*FDR* *adjusted*
	*OR (95 %CI)*	*P-*value		*OR (95 %CI)*	*P-*value		*Q-value*
WBC (×10^9^/L)	1.281 (1.168, 1.405)	< 0.001		1.301 (1.180, 1.434)	< 0.001		< 0.001
Platelet counts (×10^9^/L)	1.003 (0.999, 1.007)	0.206		1.003 (0.999, 1.007)	0.181		0.192
Neutrophil counts (×10^9^/L)	1.305 (1.190, 1.431)	< 0.001		1.319 (1.198, 1.453)	< 0.001		< 0.001
Lymphocyte counts (×10^9^/L)	0.371 (0.236, 0.582)	< 0.001		0.379 (0.238, 0.605)	< 0.001		< 0.001
Monocyte counts (×10^9^/L)	3.422 (0.914, 12.803)	0.068		4.227 (1.022, 17.478)	0.047		0.060
PLT%	4.226 (0.099, 180.563)	0.452		2.901 (0.059, 142.785)	0.592		0.592
NEU%	1.091 (1.063, 1.119)	< 0.001		1.093 (1.063, 1.124)	< 0.001		< 0.001
LYM%	0.900 (0.872, 0.929)	< 0.001		0.898 (0.868, 0.929)	< 0.001		< 0.001
MON%	0.745 (0.648, 0.856)	< 0.001		0.734 (0.634, 0.850)	< 0.001		< 0.001
NLR	1.130 (1.074, 1.188)	< 0.001		1.129 (1.073, 1.187)	< 0.001		< 0.001
MLR	11.144 (3.071, 40.437)	< 0.001		12.108 (3.338, 43.923)	< 0.001		< 0.001
PLR	1.006 (1.003, 1.008)	< 0.001		1.006 (1.003, 1.009)	< 0.001		< 0.001
SII	1.000 (1.000, 1.001)	< 0.001		1.000 (1.000, 1.001)	< 0.001		< 0.001
SIRI	1.228 (1.112, 1.357)	< 0.001		1.238 (1.121, 1.367)	< 0.001		< 0.001
LDH (U/L)	1.008 (1.003, 1.014)	0.002		1.008 (1.003, 1.013)	0.003		0.005
HBDH (U/L)	1.010 (1.002, 1.018)	0.019		1.009 (1.001, 1.018)	0.030		0.042
CK-MB (U/L)	1.002 (1.000, 1.004)	0.049		1.002 (1.000, 1.004)	0.085		0.096
CK (U/L)	1.042 (0.996, 1.089)	0.075		1.044 (0.996, 1.094)	0.076		0.091

Model 1 was crude model; model 2 adjusted for sex, age, smoking, alcohol drinking, history of hypertension, history of diabetes, and history of heart disease. *Q*-values were adjusted with Benjamini-Hochberg’s FDR correction. aSAH, aneurysmal subarachnoid hemorrhage; OR, odds ratio; WBC, White blood cell; PLT, Platelet counts; NEU, Neutrophil count; LYM, Lymphocyte counts; Mon, Monocyte counts; NLR, Neutrophil to lymphocyte ratio; MLR, Monocyte to lymphocyte ratio; PLR, Platelet to lymphocyte ratio; SII, systematic inflammation index; SIRI, systematic inflammation response index; LDH, lactate dehydrogenase; HBDH, hydroxybutyrate dehydrogenase; CK, creatine kinase; CK-MB, creatine kinase-MB, FDR, false discovery rate.

### Association between inflammatory indexes and myocardial enzymes

**Fig. 2:**
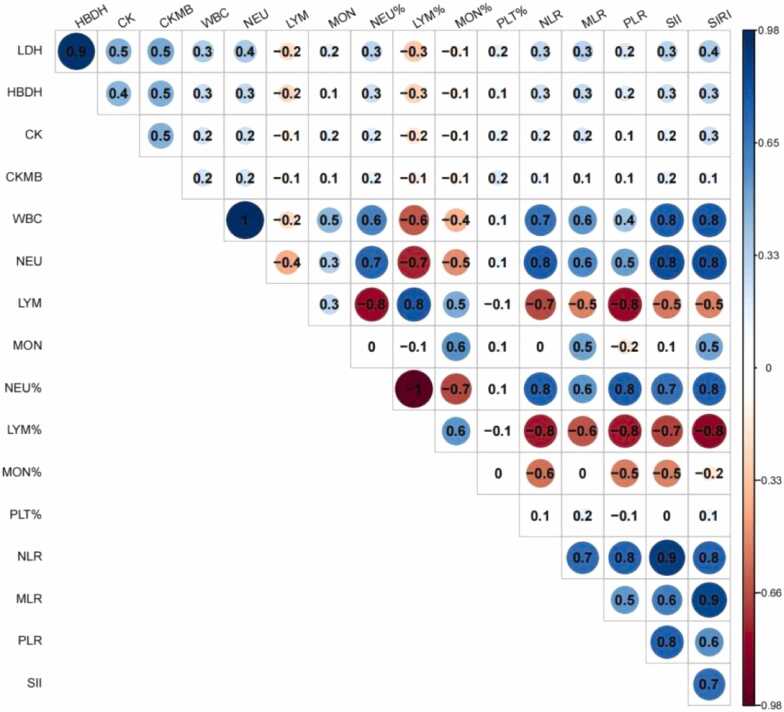
Heat map of pearson correlation coefficients between the myocardial enzymes and inflammatory indexes. Pearson correlation coefficients are represented by different colours defined in the scale on the right side of the correlation map. aSAH, aneurysmal subarachnoid hemorrhage; UIA, unruptured intracranial aneurysm; WBC, White blood cell; PLT, Platelet counts; NEU, Neutrophil count; LYM, Lymphocyte counts; MON, Monocyte counts; NLR, Neutrophil to lymphocyte ratio; MLR, Monocyte to lymphocyte ratio; PLR, Platelet to lymphocyte ratio; SII, systematic inflammation index; SIRI, systematic inflammation response index; LDH, lactate dehydrogenase; HBDH, hydroxybutyrate dehydrogenase; CK-MB, creatine kinase-MB; CK, creatine kinase.

**Fig. 3:**
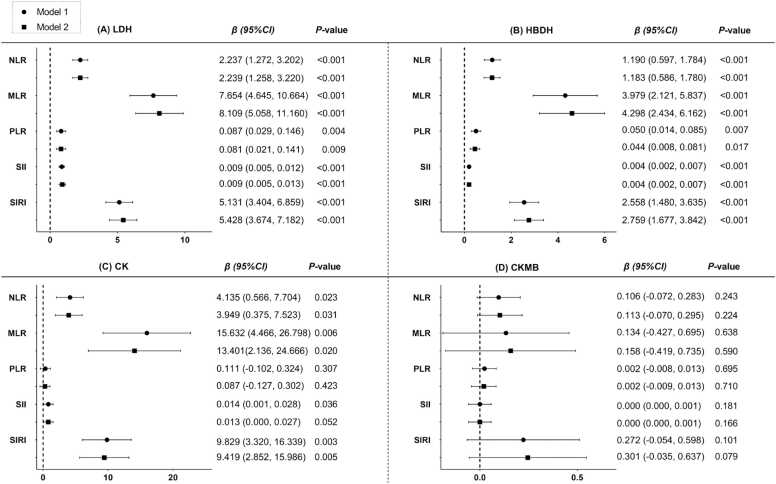
The associations between LDH (A), HBDH (B), CK (C) and CKMB (D) with inflammatory indexes. Model 1 was crude model; model 2 adjusted for sex, age, smoking, alcohol drinking, history of hypertension, history of diabetes, and history of heart disease. LDH, lactate dehydrogenase; HBDH, hydroxybutyrate dehydrogenase; CK-MB, creatine kinase-MB; CK, creatine kinase; NLR, Neutrophil to lymphocyte ratio; MLR, Monocyte to lymphocyte ratio; PLR, Platelet to lymphocyte ratio; SII, systematic inflammation index; SIRI, systematic inflammation response index.

The Pearson correlation analysis was performed between myocardial enzymes and inflammatory indexes, and the results were depicted in Fig. 2. It can be seen that there were many obvious correlations between the variables. Therefore, the linear regression analysis between myocardial enzymes and inflammatory indexes were also performed, and the results are shown in Fig. 3. The associations of LDH and HBDH with inflammatory indexes were all statistically significant in both crude and adjusted models (*P*-value < 0.05). Except for PLR, the associations of other inflammatory indexes with CK were found to be statistically significant (*P*-value < 0.05). However, no significant association was found between CK-MB and inflammatory indexes in the present analysis (*P*-value > 0.05).

## The diagnostic ability of Inflammatory index, myocardial enzyme, and all indexes for aSAH

**Fig. 4:**
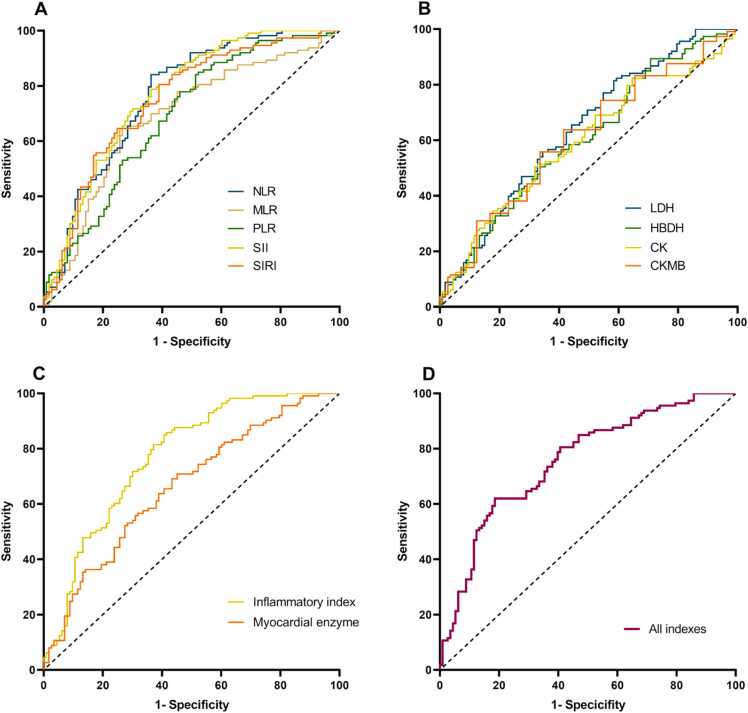
ROC curves of myocardial enzyme and inflammation levels in the identification of the patients with aSAH. LDH, lactate dehydrogenase; HBDH, hydroxybutyrate dehydrogenase; CK-MB, creatine kinase-MB; CK, creatine kinase; NLR, Neutrophil to lymphocyte ratio; MLR, Monocyte to lymphocyte ratio; PLR, Platelet to lymphocyte ratio; SII, systematic inflammation index; SIRI, systematic inflammation response index.

This study evaluated the effects of NLR, MLR, PLR, SII, and SIRI on aSAH. Univariate logistic regression analysis demonstrated that elevated levels of inflammatory indexes (NLR, MLR, PLR, SII, SIRI) and myocardial enzymes (LDH, CK) were significantly associated with the diagnosis of aSAH. Therefore, we plotted ROC curves to evaluate the diagnostic value of them for aSAH, and the results are present in Table 3 and plotted in Fig. 4. The optimal cutoff value for NLR, PLR, MLR, SII, and SIRI for detecting aSAH was 4.261 (the area under the ROC curve (AUC) = 0.765; sensitivity: 84.1 %; specificity: 63.7 %; *P*-value< 0.001), 135.784 (AUC = 0.689; sensitivity: 85.0 %; specificity: 47.8 %; *P*-value<0.001), 0.338 (AUC = 0.683; sensitivity: 64.6 %; specificity: 72.6 %; *P*-value<0.001), 1027.93 (AUC = 0.767; sensitivity: 78.8 %; specificity: 63.7 %; *P*-value<0.001), 1.676 (AUC = 0.748; sensitivity: 80.5 %; specificity: 61.1 %; *P*-value<0.001), respectively. An evaluation of the diagnostic value of the combination of five (NLR-PLR-MLR-SII-SIRI) gave an AUC of 0.771 (*P*-value < 0.001). The optimal cutoff value for LDH, HBDH, CK, and CK-MB for detecting aSAH was 175.5 (AUC = 0.637; sensitivity: 83.2 %; specificity: 38.9 %; *P*-value<0.001), 130.5 (AUC = 0.598; sensitivity: 50.4 %; specificity: 64.6 %; *P*-value = 0.011), 82.5 (AUC = 0.593; sensitivity: 50.4 %; specificity: 67.3 %; *P*-value=0.015), and 12.5 (AUC = 0.577; sensitivity: 55.8 %; specificity: 58.4 %; *P*-value=0.044), respectively. An evaluation of the diagnostic value of the combination of five (NLR-PLR-MLR-SII-SIRI) gave an AUC of 0.771 (*P*-value < 0.001). An evaluation of the diagnostic value of the combination of four (LDH-HBDH-CK-CKMB) gave an AUC of 0.662 (*P*-value<0.001). When combining both inflammatory indexes and myocardial enzymes in a single ROC curve, AUC values improved to 0.755 (*P*-value< 0.001).

**Table 3:** ROC analysis of various indicators.

Variables	AUC	*95 %CI*	*P-*value	Cutoff	Sensitivity	Specificity
NLR	0.765	0.702–0.827	< 0.001	4.261	0.841	0.637
PLR	0.689	0.620–0.757	< 0.001	135.784	0.85	0.478
MLR	0.683	0.613–0.754	< 0.001	0.338	0.646	0.726
SII	0.767	0.706–0.829	< 0.001	1027.925	0.788	0.637
SIRI	0.748	0.683–0.812	< 0.001	1.676	0.805	0.611
LDH	0.637	0.565–0.708	< 0.001	175.500	0.832	0.389
HBDH	0.598	0.525–0.672	0.011	130.500	0.504	0.646
CK	0.593	0.519–0.668	0.015	82.500	0.504	0.673
CK-MB	0.577	0.503–0.652	0.044	12.500	0.558	0.584
Inflammatory index	0.771	0.710–0.832	< 0.001			
Myocardial enzyme	0.662	0.592–0.733	< 0.001			
Inflammatory index and Myocardial enzyme	0.755	0.692–0.818	< 0.001			

ROC, receiver-operator characteristic; AUC, the area under the ROC curve; NLR, Neutrophil to lymphocyte ratio; MLR, Monocyte to lymphocyte ratio; PLR, Platelet to lymphocyte ratio; SII, systematic inflammation index; SIRI, systematic inflammation response index; LDH, lactate dehydrogenase; HBDH, hydroxybutyrate dehydrogenase; CK, creatine kinase; CK-MB, creatine kinase-MB.

Additionally, statistical comparison of AUCs using DeLong's test was conducted to substantiate improvements in diagnostic performance among the evaluated models. Pairwise comparison via DeLong's test (Table 4) demonstrated significantly superior diagnostic performance of the inflammatory index model over the myocardial enzyme model (Δ AUC=0.109, *P* = 0.012). Although the combined model showed improved efficacy versus myocardial enzymes alone (Δ AUC=-0.093, *P* = 0.002), it did not achieve statistically significant enhancement compared to the inflammatory index model (Δ AUC=0.016, *P* = 0.402).

**Table 4:** Delong's test between ROC curves of different models.

Variables	*Z*-value	Δ AUC	*P*-value
Inflammatory index vs. Myocardial enzyme	2.526	0.109	0.012
Inflammatory index vs. All indexes	0.837	0.016	0.402
Myocardial enzyme vs. All indexes	−3.152	−0.093	0.002

ROC, receiver-operator characteristic; AUC, the area under the ROC curve; Inflammatory index include NLR, PLR, MLR, SII and SIRI; Myocardial enzyme include LDH, HBDH, CK-MB and CK.

### Sensitivity analysis

Sensitivity analysis was conducted in the whole unmatched sample of 1565 participants to assess the robustness of the results. The results were consistent with the main analysis (Supplementary Table 2 and Table 5).

**Table 5:** Associations between inflammatory indexes and myocardial enzymes with aSAH for sensitivity analysis (*n=*1565).

Parameters	Model 1			Model 2	
	*OR (95 %CI)*	*P-*value		*OR (95 %CI)*	*P-*value
NLR	1.167(1.109, 1.227)	< 0.001		1.167 (1.109, 1.227)	< 0.001
MLR	20.869 (6.305, 69.067)	< 0.001		22.766 (6.78, 76.445)	< 0.001
PLR	1.005 (1.003, 1.008)	< 0.001		1.005 (1.003, 1.008)	< 0.001
SII	1.000 (1.000, 1.001)	< 0.001		1.001 (1.000, 1.001)	< 0.001
SIRI	1.325 (1.193, 1.473)	< 0.001		1.331 (1.198, 1.48)	< 0.001
LDH (U/L)	1.013 (1.008, 1.018)	< 0.001		1.013 (1.008, 1.018)	< 0.001
HBDH (U/L)	1.017 (1.009, 1.025)	< 0.001		1.017 (1.010, 1.025)	< 0.001
CK (U/L)	1.002 (1.000, 1.004)	0.027		1.002 (1.000, 1.004)	0.024
CK-MB (U/L)	1.037 (1.003, 1.074)	0.035		1.037 (1.002, 1.073)	0.036

Model 1 was crude model; model 2 adjusted for sex and age. aSAH, aneurysmal subarachnoid hemorrhage; NLR, Neutrophil to lymphocyte ratio; MLR, Monocyte to lymphocyte ratio; PLR, Platelet to lymphocyte ratio; SII, systematic inflammation index; SIRI, systematic inflammation response index; LDH, lactate dehydrogenase; HBDH, hydroxybutyrate dehydrogenase; CK, creatine kinase; CK-MB, creatine kinase-MB.

## Discussion

In this 1:1 matched case-control study, we found that a higher level of peripheral blood immune cells as well as inflammatory indexes, which indicated an increased inflammatory response, were associated with a higher risk of aSAH. Meanwhile, myocardial enzyme levels have a significant positive correlation with the inflammatory indexes, and increased levels of myocardial enzymes were also associated with a higher risk of aSAH. In addition, both inflammatory biomarkers and myocardial enzymes could serve as potential diagnostic factors for aSAH. The combined model showed improved diagnostic efficacy over myocardial enzymes alone, but no statistically significant enhancement versus the inflammatory index model.

Notably, inflammation is a key pathomechanism for the growth and rupture of IA. Previous studies found that higher inflammation levels were associated with a higher risk of RIA, and the pathophysiology of IA encompasses chronic inflammation and degradation of the vessel wall (Mirbagheri et al., 2024, Kamio et al., 2018). For example, prior investigations have indicated that elevated WBC levels in peripheral blood are suggestive of a systemic inflammatory response to acute SAH. Furthermore, the number of WBCs can be employed as an objective indicator of cerebral tissue damage, thereby enabling the prediction of the probability of subsequent secondary brain injury (Yao et al., 2018). Meanwhile, patients with IA have elevated plasma levels of neutrophil elastase, which is associated with vascular wall degradation have long been discussed (Baker et al., 1995). Compared with UIA, a higher degree of neutrophil infiltration has been observed in RIA (Frösen et al., 2004). Apart from that, many serum inflammatory biomarkers, such as CRP (Romero et al., 2014) and serum interleukin-6 (Lucke-Wold et al., 2021), were identified as risk factors for predicting an unfavorable prognosis in subarachnoid hemorrhage. In addition, several previous literature reported that NLR, MLR, and SII were crucial blood inflammatory indicators in aSAH (Shi et al., 2021, Feghali et al., 2021, Min et al., 2024). An elevated NLR was found to be associated with increased IAs stability scores, IAs growth, the size of UIAs, and even increased risk of poor outcomes (Zhang et al., 2023, Zhang et al., 2021). Similarly, a recent study investigated the relationship between circulating inflammatory indicators in patients diagnosed with symptomatic saccular UIA and asymptomatic UIA, and the findings indicated that elevated baseline NLR and LMR could be identified as independent risk factors for symptomatic UIA. Furthermore, the SII was identified as an independent predictor of aneurysmal symptoms, which may serve as a novel biomarker for the identification of symptomatic intracranial fusiform aneurysms in patients (Peng et al., 2023). Those reports in previous studies were consistent with the present study. However, the association between inflammation indexes and IA still needs further investigation. The underlying mechanisms may be as follows. First, neutrophil extracellular traps (NETs), which cause tissue damage by degrading the extracellular matrix and inducing inflammation, could promote the RIA (Korai et al., 2021). Second, an increase in inflammatory cytokines can result in the impairment of the blood-brain barrier (BBB), leading to an influx of greater numbers of immune cells and inflammatory mediators into the brain. This, in turn, can lead to the exacerbation of cerebral edema and neurological damage (Lauzier et al., 2023). Furthermore, the generation of oxidative stress and free radicals during the inflammatory process can directly damage cell membranes and intracellular structures, leading to cellular dysfunction and death. This, in turn, can contribute to the progression of aSAH (Wang et al., 2023).

IAs share similar risk factors with cardiovascular diseases, such as hypertension and smoking. Consequently, IAs may be associated with an elevated mortality rate due to cardiovascular diseases (Laukka et al., 2024). Abnormalities of the heart are a common occurrence in patients who have experienced an acute SAH (Enache et al., 2020). LDH is a glycolytic enzyme in brain tissue that may be released from damaged neuronal cells when the cell membrane is disrupted, and LDH is also a biochemical indicator of cardiac injury (Sharma et al., 2019). Prior research has indicated a potential correlation between LDH and early brain injury following aneurysm rupture. Serum LDH levels have been shown to increase with advancing H-H grades, suggesting that this may serve as a nonspecific biomarker for damaged brain tissue in the aftermath of an IA rupture (Zheng et al., 2022). The research results were similar to this study. HBDH is an oxidase enzyme that facilitates the oxidation of α-hydroxybutyric acid to α-ketobutyric acid. This enzyme is present in all human tissues, with the highest levels observed in cardiac tissues, where its activity can account for more than half of the total enzyme activity. HBDH can be a marker of cell death, reflecting in particular renal, erythrocyte, and myocardial injury (Lee et al., 2019). To the best of our knowledge, no evidence was found to suggest an association between HBDH and IA. Although no direct studies have reported a direct association between the two, given the close relationship between HBDH and tissue damage and the potential for tissue damage during the treatment of IA, further research is warranted to explore this relationship.

As a crucial enzyme involved in cellular energy metabolism, CK is widely expressed in tissues, particularly in muscle and brain tissues. CK has a tissue distribution specificity, and there are three isozymes (CK-MM, CK-MB, and CK-BB). It has been found that the degree of neurological injury and size of ruptured aneurysm are strong predictors of myocardial biomarkers elevation (Chen et al., 2022). This may be due to the increase of activation of the sympathetic nervous system and myocardial stress response caused by severe brain injury after SAH (Lee et al., 2022). The results of CK-MB were not statistically significant in the present study, probably due to the fact that it is mainly present in the myocardium rather than in the brain, and more studies on CK-BB could be conducted in subsequent studies to explore the association with brain health. Although there is limited research on the direct association between CK and IA, given the distribution of CK-BB in brain tissue, future studies may reveal a potential link between the two.

## Strengths and limitations

There were several limitations in this study. First, it was a case-control study, which led to difficulty in making a causal inference. Thus, the causal associations between inflammation and myocardial enzymes with aSAH may be difficult to interpret. Further prospective studies are required to substantiate our conclusions. Secondly, we only grouped the patients by cerebral hemorrhage, indicators like aneurysm size or aneurysm stage were not collected and analyzed in the study. Therefore, the data for assessing IA were not sufficiently comprehensive, the results obtained were not comprehensive enough, and it should be improved in future research. Thirdly, as a consequence of the fact that only a restricted number of patients testing for cardiac enzymes, resulting in a smaller sample size, so a larger sample size is required. In addition, this study identifies biomarkers associated with aSAH diagnosis but does not address their utility in predicting future rupture. Biomarkers were measured post-rupture; thus, findings pertain to diagnostic rather than predictive applications. Future prospective studies are needed to explore pre-rupture risk stratification. Furthermore, inflammatory and myocardial markers were measured at admission, but the precise interval between symptom onset and blood sampling was not recorded. Lack of control for this temporal variable could confound the findings. Despite such limitations the present study has multiple strengths. Although the causal direction of the associations cannot be determined, the matched case-control design of this study can effectively control for confounding factors. In addition, unlike in a majority of previous studies, this study have considered not only the inflammatory factors, but also myocardial enzyme indicators as potential predictors of aSAH, which further suggested the combination of the two is more promising for the prediction of aSAH.

In conclusion, the present study demonstrated that higher immune cells and their derived ratios were associated with patients with aSAH. Our results also demonstrated that using combined inflammatory biomarker values may enhance diagnostic efficiency for identifying aSAH in patients with IA. The ability of cardiac enzymes to detect aSAH was fine, but its AUC-ROC was not as great as inflammatory biomarkers. When inflammatory biomarkers are combined with cardiac enzymes, can significantly increase the latter’s predictive ability. In the future, we will refine the laboratory tests for cardiac enzymes, clinical signs, and scores of aneurysms. Furthermore, we will utilize prospective studies to elucidate the causal associations between the three and construct a simple, timely, and efficient predictive model. This model will enhance early intervention and reduce the risk of poor prognosis in these patients.

## Ethics approval and consent to participate

The study protocol was approved by the Ethics Committee of Tianjin Huanhu Hospital, and all participants provided informed consent. All procedures in this study were conducted according to the Declaration of Helsinki.

## Compliance with ethical standards

The study protocol was approved by the Ethics Committee of Tianjin Huanhu Hospital, and all participants provided informed consent. All procedures in this study were conducted according to the Declaration of Helsinki.

## Author contributions

All authors contributed to the study conception and design. Q. L. and H. W. participated in planning and designing of the study, data acquisition, analysis of the data and drafted the manuscript. H. Z., S. D. conceived of the study and performed the statistical analyses, interpreting, presenting the results and the acquisition of the data.

## Funding

This work was supported by the second batch of the Tianjin High-Level Talents Selection and Cultivation Project in the healthcare industry [grant numbers: TJSQNYXXR-D2–066] and General program of Natural Science Foundation of Xinjiang Uygur Autonomous Region [grant numbers: 2023D01C149].

## CRediT authorship contribution statement

**Hongguang Wang:** Writing – review & editing, Visualization, Resources, Methodology, Conceptualization. **Siyuan Dong:** Writing – review & editing, Methodology, Investigation, Conceptualization. **Haihong Zhang:** Writing – original draft, Methodology, Data curation, Conceptualization. **Qian Liu:** Writing – review & editing, Writing – original draft, Validation, Supervision, Software, Methodology, Formal analysis.

## Conflicts of Interest

The authors of the paper titled "Predictive value of Inflammatory Indexes combined with Myocardial enzymes for Aneurysmal Subarachnoid Hemorrhage in patients with Intracranial aneurysms" stated that the research was conducted in the absence of any commercial or financial relationships that could be construed as a potential conflict of interest.

## Data Availability

The data that support the findings of this study are available from the corresponding author, Hongguang Wang, upon reasonable request.
