# Supplementary material for: Diagnostic value of inflammatory indexes combined with myocardial enzymes for aneurysmal subarachnoid hemorrhage in patients with intracranial aneurysms
Source: IBRO Neurosci Rep. 2025 Aug 30;19:587–96. doi: 10.1016/j.ibneur.2025.08.023 (PMC12424418; doi:10.1016/j.ibneur.2025.08.023)
Supplement: Supplementary file 1 — Supplementary material [file mmc1.docx]

| Supplementary Table 1. Collinearity diagnostics of inflammatory indexes | | |
| --- | --- | --- |
| Inflammatory Indices | Tolerance | VIF |
| NLR | 0.145 | 6.877 |
| MLR | 0.238 | 4.200 |
| PLR | 0.388 | 2.578 |
| SII | 0.165 | 6.060 |
| SIRI | 0.217 | 4.607 |
| NLR, Neutrophil to lymphocyte ratio; MLR, Monocyte to lymphocyte ratio; PLR, Platelet to lymphocyte ratio; SII, systematic inflammation index; SIRI, systematic inflammation response index; VIF, variance inflation factor. | | |

| Supplementary Table 2. Demographic and clinical characteristics of the participants for sensitivity analysis (*n* = 1,565) | | | |
| --- | --- | --- | --- |
| Characteristics | aSAH | UIA | *P-*value |
| *n* (%) | 1452 (92.8) | 113 (7.2) |  |
| Age, years | 59.90 ± 11.44 | 59.41 ± 11.13 | 0.658 |
| Female, *n* (%) | 924 (63.60) | 71 (62.80) | 0.864 |
| NLR | 9.77 ± 7.22 | 5.50 ± 5.97 | <0.001 |
| MLR | 0.45 ± 0.28 | 0.32 ± 0.22 | <0.001 |
| PLR | 228.76 ± 123.33 | 175.40 ± 96.42 | <0.001 |
| SII | 2267.48 ± 1877.81 | 1259.51 ± 1490.68 | <0.001 |
| SIRI | 4.72 ± 4.92 | 2.39 ± 3.21 | <0.001 |
| LDH (U/L) | 229.54 ± 66.94 | 199.0442 ± 51.80 | <0.001 |
| HBDH (U/L) | 144.91 ± 41.62 | 128.55 ± 31.84 | <0.001 |
| CK (U/L) | 168.42 ± 431.72 | 96.55 ± 114.00 | <0.001 |
| CK-MB (U/L) | 15.31 ± 12.34 | 13.00 ± 5.61 | <0.001 |
| Continuous variables are presented as mean ± SD using the student-*t* test; categorical variables are presented as *n* (%) using the *chi*-square test. aSAH, aneurysmal subarachnoid hemorrhage; UIA, unruptured intracranial aneurysm; NLR, Neutrophil to lymphocyte ratio; MLR, Monocyte to lymphocyte ratio; PLR, Platelet to lymphocyte ratio; SII, systematic inflammation index; SIRI, systematic inflammation response index; LDH, lactate dehydrogenase; HBDH, hydroxybutyrate dehydrogenase; CK-MB, creatine kinase-MB; CK, creatine kinase. | | | |
